# Supplementary material for: Are carbon emissions trading and green financial instruments synergistic? -Comprehensive quantitative research based on content analysis
Source: PLoS One. 2024 Mar 7;19(3):e0298601. doi: 10.1371/journal.pone.0298601 (PMC10919601; doi:10.1371/journal.pone.0298601)
Supplement: S1 Data — (ZIP) [file pone.0298601.s001.zip › Data/地级市Z_F工作报告.zip!/╣π╢1⁄2/╒╪╟∞╩╨/└┤╫╘╣1⁄2╓┌║┼í╛┬φ┐╦╩2╛▌═°í┐_2003.docx]

各位代表：

　　我代表市人民政府向大会作政府工作报告，请予审议，并请政协各位委员和其他列席人员提出意见。

过去五年工作回顾

　　五年来，市政府在中共广东省委、省人民政府和中共肇庆市委的正确领导下，在市人民代表大会及其常委会和市政协的监督支持下，高举邓小平理论伟大旗帜，全面贯彻“三个代表”重要思想，以率先基本实现社会主义现代化为总目标、总任务统揽工作全局，以发展为主题，以制度创新和科技创新为动力，在历届市政府奠定的工作基础上，经受住较为复杂严峻经济形势的考验，国民经济、社会各项事业和精神文明建设取得新的成绩，顺利完成了市九届人大一次会议确定的各项任务。

　　——国民经济持续增长，质量和效益稳步提高。2002年，全市实现国内生产总值450.22亿元(当年价)，比上年增长10.3％，比1997年增长71.4％，年均递增11.4％。地方财政一般预算收入12.98亿元，按可比口径计算，比上年增长14.3％，比1997年增长97.1％，年均递增14.5％。全社会固定资产投资97.57亿元，比上年增长10.8％，比1997年增长52.2％，年均递增8.8％。再就业率提高到72.6％，城镇登记失业率控制在3％。城镇居民人均可支配收入7335元，比1997年增长31.5％，年均增长5.6％；农民人均纯收入3968元，比1997年增长13.2％，年均增长2.5％。城乡居民储蓄存款余额224.95亿元，比年初增长12.9％，比1997年增长55.1％。

　　——经济结构不断优化，产业竞争力进一步增强。二、三产业增加值在三大产业中的比重逐年增大，三大产业内部结构更加优化。

　　工业经济稳步发展。坚持工业立市的发展战略，加快发展园区经济。各级各类工业园区累计达到72个，重点开发了大旺、金渡、南江等工业园区；连续几年分别在郑州、哈尔滨、太原、昆明等地举办经贸技术洽谈会展活动，进一步提高了我市产品在国内市场的占有率；加大了科技对工业经济的推动力，高新技术产业开发区从三榕扩大到大旺经济开发区，面积从9平方公里扩展到34平方公里。高新技术产业产值占规模以上工业总产值比重由1997年的6.3％提高到2002年的11.7％。2002年全部工业企业实现工业增加值151.65亿元，比上年增长14.7％，比1997年增长61.7％，工业企业效益综合指数为127.6，比上年提高13.4个百分点，工业企业利税总额22.43亿元，比上年增长27.7％。

　　第三产业发展提速。发展了一批现代流通组织。2002年社会消费品零售总额154.62亿元，比上年增长10.6％，比1997年增长66.02％，年均递增11％。商品房销售面积和销售额分别比1997年增长1.6倍和2倍。邮电通讯事业不断发展。成功举办肇庆首届国际旅游文化节。星湖风景名胜区先后成为全国首批文明风景旅游区示范点、首家ISO14000国家级示范区和首批4A级旅游景区。2002年接待过夜旅游人数470.34万人次，旅游总收入32.85亿元，分别比上年增长10.1％和8.8％，比1997年增长56.4％和54.4％。2002年第三产业增加值158.41元，比上年增长9.2％，比1997年增长66％。

　　农业产业化水平不断提高。粮经种植比例从1997年的62.2:37.8调整为2002年的54.8:45.2，初步形成了十大农业主导产业和十大农业品牌。高要、四会、封开分别被评为全国肉桂之乡、柑桔之乡和松脂之乡。建成年销售收入500万元以上的农业龙头企业73家，带动农户30.3万户。“四通”(村村通公路、通电话、通邮政、通广播电视)工程和扶贫开发“四个ー”(解决贫困农户人均半亩“保命田”、每个农户挂靠一个龙头企业、输出一个劳动力、掌握一门实用技术)工程成效显著，切实改善了农村生产生活条件。投入5.06亿元改造农村电网，实现农村住宅到户电价0.79元/千瓦时以下的目标。2002年第一产业增加值125.04亿元，比上年增长6.9％，比1997年增长39.3％。

　　——国有企业改革稳步推进，初步建立起社会主义市场经济体制基本框架。国有企业实施了多种形式的产权改革，各县(市)区基本完成改革任务，市直改革面提高到70％。国有企业脱钩移交工作顺利完成。组建了肇通、肇联等一批资产经营(管理)公司。个体私营经济加快发展。到2002年底，全市个体私营企业注册资本金达到32.46亿元，比1997年底增长101％，个体私营企业入库税收3.58亿元，1998年以来年均递增19.83％。2002年底养老、失业保险参保人数分别比1997年底增长91.75％、101.5％。全面实施了城镇职工基本医疗保险制度。较好地落实了城乡居民最低生活保障制度。完成了新一轮政府机构改革。开展了两次行政审批制度改革，共精简各类审批事项70％。整顿规范市场经济秩序取得阶段性成果。建立了建设工程、土地和产权三大交易中心。实施了部门预算、国库集中支付和政府采购工作制度。四会市农村税费改革试点取得明显成效。

　　——对外开放力度加大，外向型经济取得新突破。2002年外贸出口和实际利用外资均创历史新高。积极实施了市场和产品多元化策略，扩大了外贸出口经营主体，完善口岸协调机制，提高了通关服务水平。2002年外贸出口总额9.01亿美元，比上年增长18.2％，比1997年增长70％。组建了行政服务中心和外商投诉处理中心，清理废止了与世贸组织规定不相符的文件规章，制定落实了招商引资政策，创新了引资方式，组织或参与了多层次、多形式的招商活动。2002年全市利用外资合同投资总额5.18亿美元，比上年增长130.9％，实际利用外资5.46亿美元，比上年增长22％，分别比1997年增长75.6％和46.4％，成功引进了韩国SK集团和美国LP集团等世界500强企业的投资项目。

　　——启动城市化发展策略，初步建立起花园式风景旅游城市基本框架。完成了城镇体系规划和部分中心镇规划的编制以及多项专项规划的升级修编。新建了江滨东路、肇庆大道、星湖大道、中调洪湖、城区污水处理厂一期工程和无害化垃圾填埋场，扩建改造了天宁路、端州四五路。广肇高速公路、马房北江公路大桥、肇庆大桥、金马大桥、德庆大桥、广宁古水大桥等一批路桥建成通车。基本完成县城通乡镇公路硬底化工程。5年共新建扩建公路2484公里。景丰联围景福段二、三期续建工程和四会、德庆、广宁城区堤路结合工程基本完成，5年累计加固堤围695公里。加强耕地整理和易地开发工作，连续4年实现耕地占补平衡。深入开展环境综合整治，政府环保目标任期责任制考核最近连续三年名列全省二类城市第一。生态环境实现良性循环，绿化率和森林覆盖率分别提高到92.9％和66.7％。深入开展花园式风景旅游城市创建活动，先后获得“中国优秀旅游城市”、“全国创建文明城市工作先进城市”、“国家园林城市”和“国家卫生城市”称号。城市化水平从第四次人口普查时的15.18％提高到第五次人口普查时的32.53％。

　　——科技综合实力进一步提高，教育事业取得新发展。形成以企业为主体的科技创新体系，90％以上大中型企业建立了不同形式的科技开发机构，200多个企业与国内科研院所开展了产学研联合。市科技中心完成征地、规划、设计等前期工作。以电子政务和教育信息化为示范工程的信息化应用水平不断提高，全市信息化综合指数达到41.07％。通过了省对我市党政班子推动科技进步考核。1998年被国家经贸委列为全国技术创新重点联系城市。1999～2000年度被国家科技部授予全国科技进步先进市称号。

　　逐步建立以县为主的基础教育管理体制，实施了中小学教育收费“一费制”，落实了免收农村困难家庭子女和城镇特困职工子女义务教育阶段书杂费政策，全面通过“普九”复查评估。基本完成薄弱学校改造计划和39所省布局调整试点示范学校建设。普通高中和中等职业技术学校在校生人数分别比1997年增长1倍多和31.8％。肇庆学院升格为本科院校，扩大了办学规模，提高了办学层次和水平。深化了教育教学改革，加强了素质教育。基本建成中小学城域网和教育资源中心。成人教育和学前教育有新的发展。社会力量办学规模不断扩大。

　　——精神文明和民主法制建设得到加强，社会各项事业全面发展。深入开展“三讲”、“两思”和“三个代表”重要思想学习教育活动，贯彻《公民道德建设实施纲要》，公民素质不断提高。全面实施“二五”依法治市规划和启动“四五”普法规划，建立完善法律援助制度。进一步健全了政府向人大及其常委会报告工作、向政协通报情况制度，逐步实现接受监督制度化、民主协商经常化。办结人大代表议案、建议437件和政协提案463件。深入推进了廉政建设和反腐败斗争，切实加强了行风建设，全面实行领导干部任期经济责任审计制度，立案查处违法违纪案件1006件。顺利完成农村村民委员会换届和城市居民委员会选举工作。加快了电子政务建设，扎实推进了市、县、镇政府政务、行政村村务和国有企业厂务公开，进一步完善了关系民生重大事项的社会听证制度。全面强化安全生产监督管理，安全生产形势基本稳定。认真做好人民群众来信来访工作。坚决打击各种严重违法犯罪活动，坚持不懈抓好与“法轮功”邪教组织的斗争，大力推进社区警务、安全文明小区和无毒社区建设，荣获“全国社会治安综合治理先进城市”称号。

　　加强文化基础设施建设，基本形成乡镇文化设施网络，成为第一个全面完成省人大“山区文化建设议案”工作的地级市。新建了群众艺术馆、博物馆、牌坊文化广场。完成重点濒危文物古迹的抢修。人口自然增长率连续四年控制在10‰以内，完成省下达的人口与计划生育各项任务。基本实现“九五”妇女儿童发展规划的主要目标。启动了卫生监督体制改革，实行了药品集中招标采购制度。连续两年被评为“全国无偿献血先进市”。农村基本实现人人享有卫生保健工作目标。完成殡葬改革计划任务。连续3届被评为省“双拥模范市”。全面启动“社区老年福利服务星光计划”。顺利完成三峡移民的接收安置工作。成功承办全国九运会肇庆赛区比赛。我市运动员参加第十四届亚运会勇夺4枚金牌。我市体育代表团在第十一届省运会上金牌总数创历史最好水平。召开了第一届世界肇庆同乡联谊大会，成立了海外交流协会，授予了首批40人荣誉市民称号，与美国库恩拉皮兹市建立了友好城市关系。文学艺术、社会科学、新闻出版进一步繁荣。市广播电视中心投入使用。审计、统计更好地为经济社会发展服务。民族、宗教、台务、侨务、档案、人防、气象等各项事业取得新发展。

　　各位代表，过去五年，我市经济社会所取得的成绩，是中共广东省委、省人民政府和中共肇庆市委正确领导，市人大支持监督和市政协参政议政，全市人民同心同德、开拓进取、努力实践“三个代表”重要思想的结果；是中国人民解放军驻肇部队、武警支队、各民主党派、人民团体、港澳台同胞、海外侨胞和各国友好人士大力支持和关心帮助的结果。在此，我谨代表肇庆市人民政府致以崇高的敬意和衷心的感谢!

　　从形势发展的要求看，我市经济社会发展整体上与珠三角发达地区相比差距仍然很大，必须急起直追，迅速扩大经济总量，大力调整优化产业结构，全面推进城市化和城镇化，尤其要加快发展民营经济，增加就业，提高农民、工人、公务员等各行各业人民群众生活水平。所有这些，都需要我们采取切实可行的措施，努力工作，务求实效。

今后五年奋斗目标和努力方向

　　今后五年是我市深入贯彻落实党的十六大精神，全面建设小康社会，率先基本实现社会主义现代化的关键时期。政府工作的指导思想是：以邓小平理论和“三个代表”重要思想为指导，以全面建设小康社会，率先基本实现社会主义现代化为总目标总任务统揽全局，以发展为第一要务，以扩大经济总量，提高经济增长质量，增强综合竞争力和整体经济素质为核心，实施工业强市、外向带动、科教兴市、可持续发展、区域协调发展五大战略，增创对外开放、科技进步、特色产业、经济体制、生态环境、发展环境六大优势，打造东、南部工业经济板块、山区经济板块和中心城区新经济板块，推进工业化、城市化、信息化和农业产业化，推动经济社会加快发展，跨越发展，协调发展，从花园式风景旅游城市向新型现代化大城市迈进，为建成宽裕小康社会，率先基本实现社会主义现代化奠定坚实基础。

　　今后五年的努力方向是:

　　——实现从花园式风景旅游城市向新型现代化大城市的新跨越。工业化、城市化、信息化和农业产业化全面推进，经济结构优化升级、城市建设现代化和国民经济社会信息化取得明显成效，城市综合竞争力和整体经济素质进一步提升，基本形成新型现代化大城市发展新格局。国内生产总值年均增长13％左右，力争到2007年比2002年翻一番。

　　——三大经济板块成为推动经济快速增长的主动力。抓好三大经济板块的规划建设。一是以高要、四会和高新技术产业开发区为主体，建设以高新区大旺工业园和四会南江、富溪、新江工业园为主的东部工业带，以市民营科技工业园和高要金渡、金利工业园为主的南部工业带，构筑起东、南部工业经济板块；二是以四个山区县为主体，通过激活山区经济资源，发展民营经济，建设一批工业园区和农业产业化基地，构筑起山区经济板块；三是在端州至鼎湖一线建设科技教育文化产业走廊，创办科技园区和大学园区，建成一批产学研相结合、研发与产业化相促进的高新技术产业基地和教育文化产业基地，构筑起中心城区新经济板块。按照城市发展定位，加强对各经济板块组织实施的指导协调，加大承接国内外产业转移的力度，力争一年打基础，三年见成效，五年大发展，尽快赶上珠三角核心区的整体发展水平。

　　——经济结构实现优化升级。新型工业化取得突破性进展。以现代物流业、现代服务业、房地产业和旅游业为主体的第三产业对经济增长的贡献明显增加。农业产业化水平进一步提高。三大产业增加值分别年均增长5％、18％和12.8％左右，到2007年，二、三产业增加值在三大产业的比重明显提高，二、三产业结构更加优化。区域经济协调发展，东、南部工业经济板块和中心城区新经济板块实现跨越式发展，缩短与珠三角发达地区的发展差距；山区经济板块提速跟进，经济结构从传统农业经济向工业经济转型。

　　——城市化跃上新水平。中心城区实现“东扩南移”，城市建成区范围扩大，城市功能进一步完善，向百万人口大城市目标迈进。构建起与珠江三角洲城市群一体化的基础设施网络。山区县城和中心镇建设提速，农村人口加速向小城镇集聚。生态建设和环境保护再上新水平。肇庆将成为珠三角城市群中令人向往、充满生机活力的城市。

　　——教育、文化等社会各项事业和精神文明建设上新台阶。建设新型现代化大城市，必须协调同步发展物质文明、精神文明和政治文明。加快构建现代终身教育体系，建设全民学习型社会，基本建成“教育强市”。历史文化名城深厚底蕴得到深入挖掘，与新型现代化大城市相适应的文化产业蓬勃发展，成为国内外知名的文化产业基地。公民素质进一步提升，社会大局保持稳定，争创“全国文明城市”。

　　——人民生活水平明显提高。就业渠道进一步扩大，人居条件得到较大改善，社会保障体系基本健全，市民多样化的物质和精神需求逐步得到满足，消费结构不断升级，人民群众的生活水平和生活质量全面提高。

　　为实现上述总体目标，新一届政府要着力打造工业化、城市化和信息化互动发展平合，推动经济社会跨越发展；着力打造园区经济带动、板块经济协调发展平台，推动经济结构优化升级；着力打造科技教育文化新经济发展平台，推动三大产业快速协调发展；着力打造现代农业产业化发展平台，推动农村经济快速发展和农民增收。重点实施十大系统工程:一是推进城区东扩南移，按照建设新型现代化大城市的要求，高起点规划，高标准建设，高效能管理，扩大城区范围，增加城市人口，优化功能布局。二是加快东、南部工业经济板块开发建设，发展以大旺、金渡、南江以及市民营科技工业园为主体的园区经济，建设成为国际级、现代化的制造业基地。三是推动山区县域经济发展壮大，促进工业园区建设上规模、上水平，实现从传统农业经济向工业经济的跨越。四是建设科技教育文化产业走廊，培育知识经济新的增长点，以龙头企业和高等院校为主体，扩大与国内外著名高等院校、科研院所和科技园区合作，建设一批科技产业园区和大学园区。五是加大基础设施建设投入，在突出抓好中心城区道路交通设施建设的同时，规划建设三水——怀集、肇庆——江门高速公路和环珠三角高速公路肇庆段；用两年时间完成景丰联围除险加固工程，建设江海直达深水港，实现基础设施现代化。六是推动国有企业改革发展，通过改革、改组、改造，做大做强国有龙头企业。七是提升千里旅游走廊内涵，开发羚羊峡自然景观和包公文化景区等一批新景区景点，建成全市一体化的大旅游格局。八是建设“教育强市”和“文化大市”。巩固基础教育，大力发展高中阶段教育和符合高新技术产业发展要求的职业技术教育，全面提升人口素质，为建设新型现代化大城市提供智力支持。深入挖掘历史文化资源，发展壮大文化产业。九是建设“数字肇庆”，推动电子政务、教育信息化等示范工程以及信息化应用体系建设，营造公开、公平、公正、高效的政务环境和良好的投资环境。十是加大环境保护建设力度，建设城区污水处理厂二期工程和各县(市)区污水处理厂，确保主要江河水质按功能达标，进一步优化自然生态环境。通过推进十大系统工程，促进全市综合竞争力和人民生活水平跃上一个新的台阶。

2003年目标任务和工作安排

　　2003年是贯彻党的十六大精神，实现“十五”时期各项目标，全面建设小康社会的重要一年。全市经济社会发展的主要预期目标是：国内生产总值增长11％左右，一、二、三产业增加值分别增长5％、14.5％和12％，全社会固定资产投资增长12％，社会消费品零售总额增长11.5％，地方财政一般预算收入增长11.5％，外贸出口总额增长12％，实际利用外资增长13％，城镇登记失业率控制在3％，人口自然增长率控制在8.8‰以下，城镇居民人均可支配收入增长4.5％，农民人均纯收入增长4.5％。

　　为实现上述今年各项工作目标，必须切实做好以下工作：

　　一、走新型工业化道路，做大做强传统优势产业和高新技术产业，提高产业竞争力

　　坚持以信息化带动工业化，以工业化促进信息化，形成科技含量高、经济效益好、资源消耗低、环境污染少、人力资源优势得到充分发挥的新型工业化发展格局。

　　运用先进适用技术特别是信息技水改造提升传统优势产业。积极利用现代信息技术零距离、扁平化、实时在线、共享共赢等特点，加快传统产业信息化进程，支持帮助传统优势产业加快建设行业技术创新中心、电子商务网站、企业管理信息系统和现代物流系统，扩大信息技术在改进工艺设施、优化生产流程、创新产品设计和强化内部管理等方面的应用。鼓励引导传统优势产业的龙头企业自主开发核心技术，培育名牌产品，增强市场竞争能力。发挥现有传统优势产业的比较优势，大力推动汽配、林化、造纸、木材加工、食品饮料、化纤、服装、制鞋、建材企业加强对外合作，扩大生产规模，形成企业集聚群体和专业化分工协作体系，提高发展水平。

　　加快发展高新技术产业。促进电子信息、生物医药、新材料等高新技术产业向集约化、规模化发展，进一步延长产业链，逐步形成具有核心竞争力的高新技术产业集群。积极实施名牌带动战略，做大做强高新技术龙头企业。充分发挥信息产业和生物制药产业拥有较强研发能力的优势，重点支持新型敏感元器件、赖氨酸等高新技术产业工程建设，力争在拥有自主知识产权的新产品产业化方面取得新突破。

　　全面提升国民经济和社会管理信息化水平。加大信息化统一规划建设力度，实现信息资源互通共享，避免因规划不力而造成的重复投资和浪费。抓紧电子政务、教育信息化等示范工程建设，提高信息化在国民经济建设、社会管理和企业中的应用水平。拓宽信息化建设的融资渠道，加快推进山区信息化，促进信息化整体协调平衡发展。

　　二、大力招商引资，全面发展民营经济，增大经济总量

　　继续办好各级工业园区。集中力量抓好大旺工业园、市民营科技工业园和金渡、南江等重点园区的开发建设。大旺园区从25平方公里扩大到100平方公里，加快建设封闭式监管加工贸易园区。市民营科技工业园实行传统产业区和高新技术产业区同步开发。强化园区社会化服务，以中心城区和各县(市)城区为载体，扩大对园区企业的物流、信息流、资金流以及生活居住等方面的服务。创新园区建设投融资机制，积极筹集资金投入园区土地开发。深化园区管理体制改革，形成“一站式”服务的运作机制。落实各项优惠政策，进一步降低园区企业的投资成本。加强对重点园区产业发展的指导，着力引进一批大项目和高新技术项目，努力形成具有竞争优势的主导产业，增强园区的综合竞争力。

　　进一步完善投资软环境。把社会管理信息化作为投资软环境建设的重要内容，加快电子政府建设，抓紧建立信息管理服务公共平台和数据库，实现政府各部门信息的互联互通，推动政府优质高效服务。加快“口岸电子执法系统”建设，完善“大通关”协调联动机制，提高通关效率。全面清理行政事业性收费，坚决取消不合理收费，严格监管公共服务价格，切实降低投资成本。加强行政服务中心建设，改革运作方式，扩大服务范围，提高服务水平。

　　提高吸收利用外资水平。进一步落实招商引资工作责任制，创新招商引资方式。大力引进国内外资本，积极承接国内发达地区的产业转移，引导鼓励现有外商投资企业增资扩产。认真配合省组织好粤台经济技术贸易交流会，发展对台经贸技术交流合作。进一步开放外商投资领域，促进外资由传统产业向高新技术产业、开发性农业和旅游业、服务业等领域拓展。大旺、金渡、南江等重点园区要大力引进投资规模大、关联带动大的项目特别是世界500强企业投资的项目。

　　毫不动摇地鼓励、支持和引导民营经济发展。按照“政治平等、政策公平、法律保障、放手发展”的方针，充分挖掘民营经济发展潜力，使民营经济成为经济的增长点和扩大就业的主渠道。成立专门管理机构，设立个体民营企业投诉处理中心，增强对民营经济发展的引导和服务。每个县(市)区都要建设1个上规模、上水平的民营工业园区。放开投资领域，降低准入门槛，放手激活本地民间资本，扩大利用发达地区民间资本。培育发展支柱产业和专业镇、专业市场，促进民营经济集优成势，集聚发展。发挥行业协会、商会和社会中介服务机构在发展民营经济中的重要作用。引导金融机构增强对民营经济的支持，鼓励具备条件的民营企业成立市场化的互助性或联合性融资担保机构开展担保业务，逐步解决民营企业融资难问题。支持骨干民营企业科技创新、制度创新和管理创新。

　　三、加快城市化进程，积极发展第三产业

　　实施城市东扩南移发展战略。加快中心城区东扩南移，推进城东新区建设，协调发展一河两岸，扩大城区范围，增加城市人口。掀起新一轮基础设施建设高潮，抓紧完善与珠三角核心区基础设施网络的规划衔接，加大以交通为重点的基础设施建设力度，抓好改造星湖大道并延伸到鼎湖城区、扩建广肇高速公路与城区连接段、开通改造端州城区主干道(端州七八路、桥北路、宋城西路、人民北路)、肇庆新港、扩建西江大桥、景丰联围除险加固等重点工程的规划设计和开工建设。积极探索实践“经营城市”的新思路和新举措，实现城市建设投资主体和融资方式的多元化，拓宽城市建设资金筹集渠道。分步编制城市控制性详细规划，稳步改造城中村。推进市政公用事业市场化、企业化改革和城市管理重心下移，加强市容环境综合治理和绿化美化工作。

　　发展现代流通业、现代服务业和房地产业。制订实施第三产业发展规划，改造传统商贸服务业，着力发展新兴第三产业。在农副产品和传统优势产品集散地扶持发展一批中高级专业批发市场，发展壮大连锁经营企业，促进传统商贸服务业加速转型。加强实物市场与虚拟市场的结合，发展为工业园区和生产企业服务的第三方物流，支持建设物流信息平台。扶持发展金融服务业、信息服务业和社区服务业。以城东新区建设为契机，引导房地产开发向智能化、生态型、社区配套的住宅小区方向发展。

　　提高旅游业发展水平。加强对旅游自然生态资源和人文景观的保护，积极引进社会各类资金投入旅游开发和旅游配套设施建设。以“大旅游”的发展思路整合全市旅游资源，加快开发羚羊峡自然景观、砚州包公文化景区、仙女湖景区、端砚文化旅游村、鼎湖山生态养生游、封开贺江生态游、广宁竹海大观、怀集温泉等新景区景点，加强七星岩旅游渡假区和高要广新农业生态园等休闲度假基地的建设，打造旅游品牌，营造旅游新亮点，进一步提高千里旅游走廊的建设水平。加大宣传推介力度，促进旅游区域合作。加快国际会展中心和五星级宾馆建设，提高旅游服务水平。

　　千方百计开拓国内外市场。继续支持企业开拓国内市场，帮助优势企业开展经贸合作。继续促进城乡用电同网同价，激活农村市场。改善消费环境，促进形成住宅、汽车、信息、教育、保健等消费热点。进一步优化出口商品和出口市场结构，发挥比较优势，大力促进机电产品、高新技术产品和有地方特色的“三高”农业产品的出口；巩固传统出口市场，努力拓展俄罗斯、中东、印度、非洲和南美等新市场。支持民营企业申领进出口经营权。推进国际质量体系认证工作，在优势产业中培育一批出口品牌，发展壮大出口龙头企业。

　　四、推进农业产业化，增大农村经济总量，增加农民收入

　　党的十六大指出“建设现代农业，发展农村经济，增加农民收入，是全面建设小康社会的重大任务”。省委要求东西两翼和山区实现跨越式发展。我市全面建设小康社会，主战场在农村，关键是加快提高农民的小康水平。

　　加大农业结构调整力度。加强分类指导，引导农民以市场为导向，以效益为中心，以科技为动力，加快粮经结构、种养结构和品种结构调整，粮经种植比例力争调整到50:50，大力发展优质、高产、高效、生态、安全农业。立足本地资源，打造特色品牌，提高传统农林产品、名优绿色食品和优质禽畜水产养殖三大类型特色农业基地的发展水平，加强农业信息化、标准化和农产品质量监督检测体系建设，做大做强10大特色农业主导产业和10大农业名牌产品，全面提高农产品市场竞争力和农业综合效益。巩固造林绿化成果，优化林种树种结构，大力推进林业产业化。

　　加快农村“三化”(城镇化、工业化、农业产业化)。抓好全市城镇发展规划纲要和中心镇规划的编制，严格依法实施。落实各项扶持政策，多渠道、多形式筹集建设资金，完善县城和中心镇基础设施建设，增强集聚二、三产业的承载力。深化户籍、教育、就业、社会保障等各项配套改革，促进农村人口向城镇集中。推动乡镇企业二次创业。搞好县城和中心镇的工业园区建设，选准产业发展突破口，有针对性地开展招商引资，放手发展非公有制经济，加快农村工业化。扶持发展一批实力雄厚、机制灵活、市场竞争能力强的农业龙头企业和产业化组织，加强农村社会化服务体系建设，运用信息技术改造发展农产品流通市场，壮大农产品加工业，提高带动农民开展产业化经营的能力，拓宽农民收入渠道。

　　深化农村各项改革。坚持以家庭联产承包责任制为基础、统分结合的双层经营体制，进一步稳定和完善土地承包经营关系，引导和规范土地承包经营权合理流转，探索农林土地资本化运营，促进土地规模经营。改革农业科技开发体制，加强农科推广应用体系建设。发展农业机械化。稳妥推进农村税费改革，清理农村各种不合理收费，加强农村财务审计监督，维护农民合法权益。

　　改善农村生产生活条件。加大农村基础设施建设力度，抓好以小型水库除险加固为主的农村水利设施建设，继续推进农村电网改造，组织实施省、县道上等级改造工程和乡镇通行政村公路硬底化工程。进一步完善生态公益林体系，广泛开展山、水、田、林、路的综合治理，深入开展创建生态示范村(镇)活动，保护发展农业生态环境，促进农业综台开发。加大扶贫开发力度，积极组织劳务输出，全面实施“百村脱贫致富”工程。建立完善农村合作医疗保障体系，落实农村最低生活保障制度。及时处理“三农”工作中的热点难点，切实解决农村生产生活用水难问题。

　　五、实施“科教兴市”战略，进一步提高科教事业的发展水平

　　加快发展科教事业，为建设科技教育文化产业走廊，增强全市经济社会发展竞争力提供强有力的智力支持。

　　大力发展教育事业。加大政府对教育的投入。完善“以县为主”的农村义务教育管理体制。加快推进中小学校布局调整，继续抓好第三批老区、山区小学改造。加强防流控辍工作，严格执行中小学收费“一费制”，巩固提高普及九年义务教育。扩大优质高中规模，加强示范性高中建设，加快启动肇庆中学高中部二期工程，鼓励发展民办高中，为普及高中阶段教育打好基础。全面推进素质教育，加强中小学信息化教育和英语教育。大力发展中等职业教育，推进布局调整和资源整合。积极发展高等教育，支持肇庆学院扩大招生规模，提高办学水平。建立多元化的办学模式和投资机制，以启动科技教育文化产业走廊建设为契机，扩大与国内外知名院校合作，建设大学园区和教育产业基地。发展继续教育、学前教育和特殊教育。培育发展远程教育，建立社区教育实验区。

　　增强科技创新能力。支持龙头企业在科技教育文化产业走廊创办信息产业科技园区和生物制药产业科技园区。深化科技体制改革，完善技术研究开发体系和面向中小企业的科技服务体系，支持开展多种形式的产学研联合，继续帮助企业组建各级各类工程技术研究开发中心。抓好市科技中心建设。鼓励发展民营科技企业，支持创办民营科技园和农业科技示范基地。加强各级生产力促进中心、技术市场和发明协会建设。切实保护知识产权。重视人才队伍建设和人力资源开发，大力引进、积极培养和放手使用高层次人才。切实改善科技人员的工作、生活条件。

　　六、扩大深化国有企业改革，进一步完善社会主义市场经济体制

　　打好国有企业改革攻坚战。积极稳妥推进国有企业改革，做到决心大、步子稳、方式新、做法活、效果实。引导重点骨干企业拓宽思路，创新体制，引入国内外同行业具有较强实力的战略投资者参与改革、改组、改造，建立完善现代企业制度。加大国有资产重组力度，组建一批规范化的股份公司争取上市。拓宽劣势企业退出通道。放开国有中小企业产权转让比例。规范产权交易行为，防止国有资产流失。妥善安置转制企业职工。支持劳动、资本、技术和管理等生产要素按贡献参与分配，试行企业经营者和技术骨干年薪制、期权制。进一步完善国有资产保值增值考核办法，建立科学合理的经营者业绩考核体系。逐步推行国有企业经理人员公开选拔制度。

　　深化各项改革。按照中央和省的部署，深化国有资产管理体制改革。完善社会化中介服务体系，鼓励和引导国内外知名的中介机构进入我市，促进信息、咨询、会计、法律、评估等中介服务业的良性竞争和规范发展。鼓励发展要素市场，进一步规范完善有形建筑市场、有形土地市场和国有产权交易市场。深入整顿规范市场经济秩序，组织打击和防范各类经济犯罪的专项斗争。加快建立社会信用体系，抓好重点行业、重点部门的道德诚信建设，开展“信用镇、村”建设试点。

　　七、加强国土管理和环境资源保护，促进环境与经济社会协调发展

　　增强国土资源对可持续发展的保障能力。坚持把国土资源放到可持续发展大局中统筹管理，建立对国土资源严格依法保护与合理开发使用相结合的机制。切实加强耕地保护、土地用途管制，合理安排非农林用地，优化用地结构。组建土地储备中心，增强政府调控土地的能力。抓好耕地储备工作，确保耕地占补平衡。

　　扎实推进环境保护和生态建设。进一步落实各级政府环境保护目标责任制，实现创建国家环保模范城市目标。加大治理星湖和西江水质的力度。强化大气环境质量保护。加强固体废弃物综合整治。严格建设项目环保审批。切实治理污染严重的企业。大力推行清洁生产，推广环保实用技术，发展环保产业。做好全市生态建设规划和生态功能区划，加强对自然保护区、风景名胜区的管理，加大对森林公园、生态公益林、水源涵养林、生物防火林带工程的建设管理力度。依法保护和合理开发自然资源，健全国有资源有偿使用制度。加强对矿山、石场的环境管理。健全环境监测体系，加强地质环境保护和气象预测预报工作，提高抵御自然灾害的能力。

　　八、积极推进社会保障和就业工作，全面提高人民生活水平

　　加强社会保障体系建设。依法扩大社会保险覆盖面，继续强化社会保险费征缴工作，加大清收欠费力度。推进城镇职工基本医疗保险制度改革，确保年内覆盖全市。提高社会保险统筹层次，年中完成中心区社会保险统筹，年底完成全市社会保险统筹。按时足额发放离退休人员养老金。对符合最低生活保障条件的城乡居民做到应保尽保。多渠道筹集经济补偿资金，理顺下岗职工劳动关系。

　　做好就业和再就业工作。认真落实各级党委、政府制定的促进就业和再就业的各项政策措施。加快发展劳动密集型产业，培育发展物业管理、家政服务、保洁绿化等新兴服务业，挖掘就业潜力，拓宽就业渠道。完善劳动力市场建设，建成各县(市)区劳动力市场信息网并加快与省、市及发达地区联网。

　　切实解决困难群众生产生活问题。以解决困难群众“四难”(住房难、子女上学难、就医难和打官司难)问题为重点，加大投入，帮助困难群众解决生产、生活困难。适当提高最低生活保障标准和重点优抚对象生活、医疗补助标准。切实做好救灾救济工作。继续落实农村困难家庭子女义务教育阶段免收书杂费和城镇特困家庭子女减免书杂费政策。加快建立公职律师事务所，拓宽法律援助渠道。

　　九、加强先进文化和民主法制建设，发展社会各项事业

　　深入开展精神文明建设活动。围绕建设“全国文明城市”的目标，广泛深入开展城乡文明创建活动，创建一批文明城市、文明示范中心镇、文明示范社区、文明示范窗口、文明示范旅游景区。加强思想道德建设，贯彻《公民道德建设实施纲要》，重点抓好市民素质的提高和良好社会风气的形成。

　　积极发展文化事业和文化产业。制定实施文化事业和文化产业发展规划，加快文化管理体制改革，加强文化行业管理，加大文物保护开发力度，发展与国家级历史文化名城、优秀旅游城市相协调，与广大人民群众日益增长的文化需求相适应的文化产业体系，使文化产业成为新的经济增长点。以社区和乡镇为重点，加强文化阵地和文化队伍建设。进一步繁荣文艺创作，培育具有地方特色的优秀艺术精品。

　　推进民主法制建设。进一步拓宽政府与人大、政协沟通渠道，坚持政府向人大及其常委会报告工作、向政协通报情况的制度，认真办好人大代表议案、建议和政协提案，自觉接受人大监督，发挥政协和各民主党派、人民团体的民主协商、参政议政作用。依法健全政务公开、厂务公开、村务公开制度，完善村民自治、城市居民自治和企事业单位以职工代表大会为基本形式的基层民主管理。继续完善关系民生重大事项的社会听证制度。认真抓好“四五”普法教育，努力提高政府依法行政水平和公民法律素质。

　　提高人口与计划生育工作水平。认真宣传、贯彻、落实《中华人民共和国人口与计划生育法》和《广东省人口与计划生育条例》，加大计生执法力度。坚持和完善人口与计划生育目标管理责任制，坚决完成省下达的人口与计划生育任务。继续推进计划生育工作重心下移。加快计划生育信息化建设。

　　促进社会各项事业发展。继续深化卫生体制改革，全面实施城镇医疗机构分类核定、分别管理制度，逐步实现卫生院站一体化管理，巩固完善农村三级医疗卫生服务网络，初步建立起社区卫生服务框架。规范完善医疗机构药品集中招标采购制度，严格医疗收费管理。进一步加强流行性疾病控制、预防保健和爱国卫生工作，切实有效控制非典型肺炎疫情。加快中医院建设和中医人才的培养。继续做好无偿献血工作。严格监管医疗市场，继续深入整顿与人民健康密切相关的食品、药品等市场。加强体育场馆设施建设管理，深入实施《全民健身计划纲要》和《奥运争光计划纲要》，努力构建全民健身服务保障体系。办好市第七届运动会。鼓励扶持发展体育产业。加强审计和统计工作。抓好全民国防教育，加强城市人防建设，做好双拥共建和优抚安置。推进社区建设。依法管理民间组织。切实做好殡葬改革。认真实施妇女、儿童发展规划。进一步发展民族、宗教、老龄、残疾人、档案、气象、地方志等社会各项事业。

　　十、建设廉洁勤政务实高效政府，优化发展环境

　　加快政府职能转变。继续推进政资分开、政企分开、政事分开和政社分开，强化政府公共服务职能。健全政务公开机制，推进电子政府建设，促进政务公开信息化、规范化、制度化。进一步改革行政审批制度，精简审批事项，减少审批环节，规范审批行为。深化行政执法体制改革，落实行政执法责任制，开展综合行政执法试点，积极探索行政执法评议考核和行政执法监督的新思路、新措施。

　　加强政风建设和廉政建设。按照“三个代表”的要求，进一步转变机关作风，强化公务员的服务意识，提高政府行政效能和服务质量。优化公务员队伍结构，提高公务员整体素质。认真落实党风廉政建设责任制，切实加强对各种权力的监督，从源头上预防和治理腐败。继续纠正部门和行业不正之风，着力解决群众反映的突出问题。

　　进一步完善公共财政体制基本框架。改革城区财税体制。全面实施部门预算，落实“收支两条线”制度。规范财政资金供给范围，推进国库单一帐户制度试点，加快国库集中收付制度改革。

　　切实维护社会稳定。落实社会治安综合治理目标管理责任制，深入开展专项严打整治行动，完善社会治安综合治理长效机制，严密防范各种敌对分子和“法轮功”邪教组织的破坏活动，加大禁毒工作力度，落实防范和处置群体性事件的联络工作机制，及时排查化解人民内部矛盾，切实维护社会长治久安。加强安全生产监督管理，层级落实安全生产责任制和特、重大安全事故行政责任追究制度，指导、监督企业抓好安全生产管理和安全基础建设，全面推行注册安全主任制度，重点开展对非煤矿山、道路和水上交通、危险化学品、公众聚集场所消防安全等专项整治，努力遏制安全生产事故发生。

　　各位代表！过去五年，顺利完成了市九届人民代表大会确定的各项目标任务和“九五”计划，为实施“十五”计划开创了新的局面。在全面建设小康社会新的发展阶段，在以胡锦涛同志为总书记的党中央领导下，在党的十六大精神的指引下，只要我们继续高举邓小平理论伟大旗帜，深入贯彻“三个代表”重要思想；只要我们充分发挥、全面调动全市上下的聪明才智和积极性；只要我们团结一心、奋发图强、加倍努力，肇庆人民渴望追赶珠三角经济发达地区，推动经济社会跨越式发展的目标就一定能够达到，建设新型现代化大城市的宏伟蓝图就一定能够实现！
